# Physicians' Response and Preparedness of Terrorism-Related Disaster Events in Quetta City, Pakistan: A Qualitative Inquiry

**DOI:** 10.3389/fpubh.2022.912762

**Published:** 2022-06-27

**Authors:** Fazal Ur Rehman Khilji, Zil-e-Huma, Nosheen Sikander Baloch, Maryam Shoaib, Zaffar Iqbal, Abdul Raziq, Nabila Sadaf, Syed Ainuddin, Sajjad Haider, Fahad Saleem, Qaiser Iqbal, Tanveer Hussain, Asfandyar Ayaz, Rabia Ishaq

**Affiliations:** ^1^Faculty of Pharmacy and Health Sciences, University of Baluchistan, Quetta, Pakistan; ^2^Department of Zoology, Sardar Bahadur Khan Women's University, Quetta, Pakistan; ^3^Department of Gynecology and Obstetrics, Bolan Medical College, Quetta, Pakistan; ^4^Department of Gynecology and Obstetrics, Sandeman Provincial Hospital, Quetta, Pakistan; ^5^Health Department Government of Balochistan, Quetta, Pakistan; ^6^Department of Statistics, University of Balochistan Quetta, Quetta, Pakistan; ^7^Department of Pharmacy, Sardar Bahadur Khan Womens' University, Quetta, Pakistan; ^8^Department of Disaster Management and Development Studies, University of Balochistan, Quetta, Pakistan

**Keywords:** physicians, response, preparedness, terrorism-related disaster events, qualitative

## Abstract

**Background:**

Besides catastrophes, infrastructural damages, and psychosocial distress, terrorism also imposes an unexpected burden on healthcare services. Considerably, adequately-prepared and responsive healthcare professionals affirms effective management of terrorism-related incidences. Accordingly, the present study aimed to evaluate physicians' preparedness and response toward terrorism-related disaster events in Quetta city, Pakistan.

**Methods:**

A qualitative design was adopted. Physicians practicing at the Trauma Center of Sandeman Provincial Hospital (SPH), Quetta, were approached for the study. We conducted in-depth interviews; all interviews were audio-taped, transcribed verbatim, and analyzed for thematic contents by a standard content analysis framework.

**Results:**

Fifteen physicians were interviewed. The saturation was achieved at the 13th interview however we conducted another two to validate the saturation. The thematic content analysis revealed five themes and 11 subthemes. All physicians have experienced, responded to, and managed terrorism-related disaster events. They were prepared professionally and psychologically in dealing with a terrorism-related disaster. Physicians identified lack of disaster-related curricula and training, absence of a standardized protocol, recurrence of the disaster, and hostile behavior of victim's attendants during an emergency as critical barriers to effective terrorism-related disaster management. Among limitations, all respondents mentioned workspace, and resources as a foremost constraint while managing a terrorism-related disaster event.

**Conclusion:**

Although physicians understood the abilities and had the required competencies to mitigate a terrorism-related disaster, lack of workspace and resources were identified as a potential barrier to effective disaster management. Based on the results, we propose reconsideration and integration of the medical curriculum, particularly for terrorism-related disaster management, collaboration, and communication among various stakeholders to manage terrorism-related disaster events competently.

## Introduction

Defined as “*an unexpected and devastating event that exceed the coping capacity of the affected community*,” disasters have existed throughout the human history (1, 2). Although, the precise prevalence of disasters is not quantifiable, it is believed that disasters occur every day somewhere in the world (3, 4). Shifting our concerns to disasters in the South Asian Region, the past 40 years have reported 1,333 disasters which resulted in 980,000 deaths. The disasters also affected 2.4 billion lives and was worth US$ 105 billion of overall damages (5).

Parallel to the natural disasters, mankind is also faced with terrorism-related disasters (TRDs) that are a rampant challenge across the globe (6). The Global Terrorism Database (GTD) defines TRDs as “*the threatened or actual use of illegal force and violence by a non-state actor to attain a political, economic, religious, or social goal through fear, coercion, or intimidation”* (7). Terrorism is believed to be a leading menace to humanity (8), and since the 1970's, a rising trend of TRDs has been reported (9). Around the globe, GTD reported 156,772 TRDs from 1970 to 2015 (10). On an average of 2,000 TRD events per year reported, most attacks were executed in the Middle East and South Asia (11), which included Afghanistan and Pakistan (12).

Shifting our concerns toward Pakistan, terrorism in the country mainly originated in the Soviet-Afghan war in the 1980s (7). The September 11 attacks entirely transformed the panorama of terrorism throughout the world (13). Because of the strategic location of the country and its influential capacity regarding Afghanistan, the country is immensely affected by TRDs, and it consequently fuelled terrorism to its extreme (14–16). According to Global Terrorism Index (GTI), Pakistan was ranked 2nd in terms of 1,650 terrorist attacks, surpassing Afghanistan with 1,468 terrorist attacks in 2012. However, since then a declining trend was observed as in 2017 Pakistan counted for 7% of TRDs around the globe and further decreasing to only four attacks in 2021 (15). Terrorism is not limited to cause massive catastrophes and casualties only, but additionally, it imposes adverse effects on the financial market and economic condition globally (17). The economic growth decreases with the increase of TRDs (18) as there is an adverse relationship between Foreign Direct Investment (FDI) and terrorism (19). Correspondingly in Pakistan, the cost of counter-terrorism was US$ 2.669 billion that increased to US$ 17.8 billion during 2010–2011. Thus, resulted in a decrease in the investment to Gross Domestic Product (GDP) ratio and FDI inflows in the country (20). Furthermore, to combat terrorism-related activities, the Government expenditures augmented from 7.84% of GDP in the year 2005 to 11.84% of GDP in the year 2015 (7). Resultingly, this shift of resources from the productive sectors to security-related activities made the already deprived economic condition more vulnerable (21).

In addition to economical destructions and demolitions, TRDs induce significant impacts on a health care system's capacity to act proficiently and respond effectively (22). This is true as the public healthcare system in Pakistan is scarce in terms of human resources and substantial infrastructure to accomplish the requirements of the growing population (23). According to World Health Organization (WHO), Pakistan' healthcare system lacks basic healthcare guidelines, bears the sixth minimum health expenditure to GDP ratio, and acquires only 0.6 hospital beds for every 1,000 people (24). Specifically talking about managing TRDS, research reveals that Pakistan has not adopted the recent advancements in scientific approaches of disaster management (DM) policies (25). Currently, Pakistan lacks an effective response planning system and valuable resource allocation system during TRDs (26). The higher death ratio in Pakistan during TRDs are attributed to the amplitude of collapse, lack of structured emergency medical services, increased infection rates, inadequate triaging, and deficiency in primary resuscitation services (27). Summarizing, the healthcare system of Pakistan is confronted with an overburden of TRDs upon its occurrences. Under such circumstances, it is important that health institutions and healthcare professionals are proficient, prepared, and skilled to administrate a terrorism-related incidence with restricted resources otherwise the defiantly consequences might be significantly vulnerable. Additionally, acknowledgment and consideration of preparedness and administration are imperative to overcome future disasters effectively. However, to the best of our knowledge and through extensive literature review, healthcare professionals' preparedness and response to TRDs in Pakistan is not reported in the literature. Considering the high incidence of terrorism-related events in Pakistan and scarcity of information on its management, we designed this study to evaluate the response and preparedness of healthcare professionals (physicians) toward terrorism-related events in Quetta city, Pakistan.

## Methods

### Study Design and Settings

We adopted a qualitative study design (in-depth, face-to-face interviews). The flexibility of this method, including the exhaustive analysis of respondents' attitudes, experiences, and intentions, is beneficial (28, 29). Similarly, qualitative studies produce an extended range of ideas and notions to which persons carry out issues; also, the different point of view is revealed among the groups (30, 31). It is imperative to mention that the qualitative method has the edge to fill the gaps that remained undiscovered by using research-based surveys, specifically in underdetermination areas of the research (32). Subsequently, considering the objective aspects of the current study, a qualitative design was a paramount adoption for preliminary essence anticipated to generate concepts and hypothesis that bears added potential for research compared to other models (33).

For the current study, the phenomenological approach was adopted. The rational of adopting this approach was straight forward. Phenomenological approach in qualitative methods emphasizes experiential, lived aspects of a particular construct—that is, how the phenomenon is experienced at the time that it occurs, rather than what is thought about this experience, or the meaning ascribed to it subsequently (29).

The current study was conducted at Trauma Center Quetta (TC). The TC was established in 2016 within the premises of Sandeman Provincial Hospital Quetta (SPH-Q). The TC is a thirty-bedded facility with 24 physicians stationed to offer healthcare services that are trauma specific. The TC is well-equipped and bears the capacity to deal with all kinds of emergency conditions. The TC also provides immediate health care services round the clock and throughout the year to the victims of terrorism-related and general traumatic injures (34).

### Study Participants, Criteria, and Sampling

Registered physicians stationed permanently and practicing at the TC were approached for the interviews. Our objectives made it apparent to adopt the purposive sampling method (35). We excluded physicians on rotations, stationed at the TC as part-timers and unwilling to participate. For content analysis, we opted the saturation based approach. The theoretical saturation means that no additional data is retrievable, and the researcher can develop properties of the category. Furthermore, as same inference occurs over and over again, the researcher becomes empirically confident that a category is saturated (29).

### The Interview Guide (Validation, Reliability, and Pilot Study)

A semi-structured interview guide was used for the interviews. The guide was constructed through extensive literature review (36–40), expert panel discussion, and shared experience (41–43). The guide was established with widely framed, open-ended questions that gave enough opportunities to the respondents. Parallel, physicians were provided the opportunity to illustrate their narratives and encourage sharing additional information regarding terrorism-related disaster management.

The face and content validity of the interview guide was established through a panel of experts (senior physicians) having experience in terrorism-related disaster management. Once the validity was ensured, the guide was piloted with four physicians. The pilot data analysis confirmed that the discussion topics were adequate and appropriately phrased to answer research questions and minimize validity and reliability threats. As the validity and reliability of the interview guide was ensured, it was made available for the main study (Supplementary File). Data and participants of the pilot study were not included in the final analysis.

### Interview Procedure, Data Collection, and Analysis

The first author conducted the interviews at the TC; all participants were briefed about the study objectives before initiating the interviews. Continuously, a debriefing session was again conducted at the end of the discussion. The interviews started with an ice-breaking session. Probing questions were asked in conversations to clarify the meanings of responses and gain insight into the topic being discussed.

All interviews were audio-recorded that persisted for approximately half an hour. Along with eliciting in-depth views, the physicians were also provided the freedom to express the additional reviews and comments. During the interviews, ZI played a role as an observer while AR assisted in monitoring the field notes, facial expressions, and body language that were concomitant to audio recordings. The conducted interviews remained until the achievement of thematic saturation (44, 45). The analysis of recordings (verbatim) was carried out by the research team, followed by an informal gathering where physicians were demonstrated with finalized interview script (46). Triangulation and member check helped in establishing the credibility and contributed to trustworthiness. Parallel to triangulation, persistent observations by the research team, transferability of the data and confirmability approached were used to enhance the trustworthiness and credibility of the data analysis.

Later, physicians were asked for affirmation of correctness and clarity of words, ideas, and jargon used during the script analysis (47, 48). NVivo® was used for coding and analysis through iterations (49) and inconsistencies were cleared through mutual consensus. All emerging themes and subthemes were discussed for authenticity among the research team and presented for data inference and interpretation. Although there are various approaches to conducting thematic analysis, we adopted the process developed Braun and Clarke that followed a six-step process: familiarization, coding, generating themes, reviewing themes, defining, and naming themes, and writing up.

### Ethical Approval

Institutional review board at the Faculty of Pharmacy & Health Sciences, University of Baluchistan approved the study protocol (UoB/Reg:/GSO/67). Written consent was taken from the respondents before the interviews. The physicians were introduced to the nature of the research prior to initiating the interviews. They were also made secure of the confidentiality of their responses and their right to withdraw from the study.

## Results

### Demographic Characteristics of Physicians

The saturation was achieved on the 13th interview, but we conducted two additional interviews to validate the saturation. Fifteen physicians were interviewed out of those 9 (60%) were male. Six (40%) of the respondents had an overall experience of >10 years and 7 (46.7%) had experience of > 18 months at the TC. Eleven (73.3%) had specialization in surgery and none of the physicians were trained precisely in terms of managing or responding to disasters as shown in Table 1.

**Table 1:** Demographic characteristics of the respondents.

**Characteristics**	**Frequency**	**Percentage**
**Age (years)**		
26–35	10	66.7
36–54	5	33.3
**Gender**		
Male	9	60.0
Female	6	40.0
**Education**		
MBBS	11	73.3
FCPS	04	26.7
**Overall experience (years)**		
1–5	7	46.7
6–10	2	13.3
>10	6	40.0
**Experience at trauma center (months)**		
6–12	7	46.7
13–18	1	6.7
>18	7	46.7
***Current position***		
MO	11	73.3
Registrar	1	6.7
PG registrar	2	13.3
Consultant	1	6.7
**Specialization in disaster management**		
None	15	100

Thematic content analysis resulted in five major themes and 11 subthemes (Table 2). The themes and sub-themes are discussed as under:

**Table 2:** Themes and subthemes identified during data analysis.

**Themes**	**Subthemes**
**Theme 1**: Terrorism-related disaster event (experience, information source	**Subtheme 1(a)**: Experience of terrorism-related disaster event(s)
and call-up mechanism)	**Subtheme 1 (b)**: Disaster-related information sources and call-up mechanism
**Theme 2**: Response toward terrorism-based disaster event	**Subtheme 2(a):** Professional response
	**Subtheme 2(b):** Personal response
	**Subtheme 2(c):** Inclusive response
**Theme 3:** Preparedness of terrorism-related disaster	**Subtheme 3(a):** Current level of knowledge and familiarity of terrorism-related disaster management
	**Subtheme 3(b):** Workforce, infrastructure, and supplies
	**Subtheme 3(c):** Triage, communication, and coordination
**Theme 4:** Barriers toward terrorism-related disaster	**Subtheme 4(a):** Safety concerns and issues
management	**Subtheme 4(b):** Lack of disaster management content
	**Subtheme 4(c):** Lack of drills and hands-on training
**Theme 5:** Suggestions and recommendations	

#### Theme I: Terrorism-Related Disaster Event (Experience, Information Source, and Call-Up Mechanism)

##### Subtheme 1(a): Experience of Terrorism-Related Disaster Event(s)

All physicians reported experience of improvised explosive devices (IEDs), suicide attacks, mass shooting attempts on security forces, law enforcement agencies, and civilian populations. The suicide incidence of August 2016 was specifically mentioned, which occurred within SPH-Q consequent to 50 deaths.

***“I was at TC when a pronounced lawyer (injured) victim of the targeted shooting was shifted to the emergency department (ED). A crowd of lawyers gathered at ED. After a little while, oh God! (appearing deep grief on his face) a tragic bomb blast occurred at the entrance of ED. Indescribable abhorrent scene converted the hospital not less than into a battlefield. After that, it was declared a suicide attack after recovering the dead body of suicide bomber.” (Physician 2, Male)***

Another male physician (Physician 6) commented that:

***“I cannot forget the horrible incident of bomb blast followed by the mass shooting at Bethel Memorial Methodist Church in the city. It was a challenging condition for all of us.”***

##### Subtheme 1(b): Disaster-Related Information Sources and Call-Up Mechanism

For an efficient and effective response to a disaster significantly, healthcare practitioners should promptly be informed immediately after the occurrence of a disaster. Therefore, an updated, sound, and efficient information system to respond is an essential requirement for communication across the hospital and other affiliated institutes and allied agencies. For that reason, physicians were inquired about the source of information and call-up mechanism in their institute during a terrorism related disaster event.

***“We have an appropriate institutional/departmental WhatsApp group where exclusive and updated news and events are shared frequently. Moreover, national/International news networks are also a source of information regarding terrorism-related events. Likewise, Quetta is a small city soon after the occurrence of a grievous incidence; the information spreads within no time.” (Physician 3, Female)***

We asked the respondents regarding the call-up mechanism contingent upon terrorism-related disaster emergencies. The hospital administration informs all physicians of the TC about the terrorism-related incidence. Additionally, physicians are liable to report at TC within no time once they receive information about untoward news through any source. The study respondents appreciated the information and call-up system adopted by the institute in contacting the physicians during an emergent condition.

***“The duty schedule is designed based on eight hourly rotational shifts; therefore, one shift always remains present on duty at TC. Off duty physicians are informed through a phone call. The mechanism of reporting is simply reasonable and useful.” (Physician 9, Male)***

### Theme 2: Response Toward the Terrorism-Based Disaster Event

#### Subtheme 2(a): Professional Response

The response comprises practices and actions engaged in mitigation of concise and extended effects of a disaster. A quick and efficient response ensures instant assistance in saving precious lives, adequate medical therapy, and minimizing the collective impact of the disaster. Therefore, the comprehension response of healthcare professionals is a matter of great concern through the disaster. Relating to the responses we received, physicians were confident to respond to such terrorism-related disasters professionally and proficiently. Based on their disaster management experiences and the frequency of events they came across, according to our respondents, they feel prepared to respond to an emergent event appropriately within time.

***“As soon as we receive an emergency call (or get information from any means), we ensure and supervise the arrangements along with organizing necessary equipment and allied resources at TC. We also ensure a quick check of the operation theaters, intensive care unit, wards beds, and accessibility of medicine trolleys. We provide necessary expert opinions to fulfill the gaps before the arrival of the victims.” (Physician 10, Female)***

#### Subtheme 2(b): Personal Response

In responding to a disaster, facing chaos and panic situations is a natural phenomenon. Healthcare professionals remain at risk of being exposed to trauma, which results in post-traumatic anxiety, stress, and depression. Such circumstances may lead to affect their psychosocial well-being adversely. Therefore, it is essential to understand the psychological response of physicians in such intimidating situations. Following what is being described, physicians of the current study showed a positive response, willpower, and willingness to lead to driving the team during responding and managing victims brought to the TC.

***“When the victims arrive at the TC, the situation and atmosphere is indescribable. Therefore, we must lead the other healthcare professionals, allied staff, victims, and attendees of the victims professionally and ethically. We must manage the stressed and chaotic situation, and for that, we are always ready … physically and emotionally.” (Physician 4, Male)***

#### Subtheme 2(c): Inclusive Response

Even though our study respondents were prepared, certain deficiencies were also noted during the interviews. Like other developing countries, the healthcare system of Pakistan is deprived and faced with the nonexistence of a disaster management system and response mechanism. For that reason, the physicians' response to emergencies is based on their self-experiences of countering numerous terror attacks.

***“The emergency response checklists and smart response systems are theoretically familiar words. In my opinion, adopting and establishing a profound system at TC regarding response to disasters can be a great support for us. Currently, we are managing the situations on self-experiences basis without having specific operating guidelines.” (Physician 5, Male)***

### Theme 3: Preparedness of Terrorism-Related Disaster Management

#### Subtheme 3(a): The Current Level of Knowledge and Familiarity of Terrorism-Related Disaster Management

Preparedness is the process of considering the potential occurrence of disaster and taking necessary action regarding filling the gaps before a disastrous event. The period of peace is an excellent opportunity to be utilized for preparation, integrated planning, organizing resources, and designing of procedures for the imminent threats and risks of disaster consequent to minimize its impacts. Evaluating the knowledge of DM among healthcare professionals is an imperative to reference to disaster-related uncertainties. During the interviews, physicians of the current study articulated sound reservations relevant to their knowledge and awareness of terrorism-related disaster management. Additionally, all the respondents confirmed that there are no training opportunities available, nor any seminars/workshops arranged that can help in improving their perceptive and knowledge of terrorism-related disaster management. In furtherance, they also stated disaster-related drills and mock exercises are beyond their imaginations.

***“It is my fourth year working at TC. Since then, I have not received any formal training about terrorism-related disaster management. There is no documented informational module or written material that can help in improving relevant knowledge. We are managing terrorism-related disaster emergencies purely based on self-experiences gained from multiple encounters of terrorism-related disasters.” (Physician 14, male)***

#### Subtheme 3(b): Workforce, Infrastructure, and Supplies

The existence of a disaster management framework is another critical aspect while discussing disaster preparedness. Nevertheless, Pakistan is confronted with a poor disaster management framework, scarce health budgets, shortage of specialized healthcare professionals, and an overburdened healthcare system. Our study respondents expressed similar views when they were enquired about the availability of workforce, organizational framework, and infrastructure at TC. Though, the respondents of our study were satisfied with the medicine and supplies to some extent that are available at the TC.

***“Comparative to the frequency of TRDS, we are deficient in terms of the workforce at TC. Similarly, the space is also limited. During terrorist attacks of mass casualties, we must utilize other sections of the hospital, even must shift the victims to other institutes. We do not hesitate to perform additional duties and overload ourselves because we are dedicated in our motive to saving lives on a priority basis even with limited resources.” (Physician 1, Male)***

During the discussion, Physician 12 (Female) added that:

***“To some extent availability of medicine is satisfactory. The medicine trolleys are well prepared by pharmacists before any unwanted terrorism-related incidence. In my opinion, it may be enough to handle 80-90 victims of such event.”***

#### Subtheme 3(c): Triage, Communication, and Coordination

Triage is the process of prioritization (order of treatment) during mass disaster events. An effective triage depends on a functional and integrated command and communication system which enables in the identification of treatment priorities during the management of mass emergencies. Surprisingly, our respondents had limited knowledge of treatment orders because of poor knowledge of disaster management (as discussed above) and lack of planning and coordination within hospital departments and among different institutes.

***“Triage! (Confused), soon after receiving the victims, we provide first aid and manage them according to their needs and severity. We have no protocols determinate to the treatment order. Frankly speaking, tirage is known to me, but I have no idea about its appropriate utilization.” (Physician 8, Male)***

Our study respondents also reported issues regarding lack of structured coordination and updated communication system. Within the TC, a traditionally acceptable coordination and communication system is existing, while; physicians had some reservations about other departments of the institute.

***“Patients are brought to casualty department or simultaneously to the TC in routine days. This results in a delay while managing the victims along with confusion in terms of actualization the number of casualties and prioritization (order of treatment). We can overcome such conditions through effective coordination and communication.” (Physician 11, Female)***

### Theme 4: Barriers Toward Terrorism-Related Disaster Management

#### Subtheme 4(a): Safety Concerns and Issues

The chaotic and panic environment, crowd, recurrence of the terror attack at hospital setting and hostile behavior of the victim's attendees during an emergency was mentioned as a key barrier toward terrorism-related disaster management. Our respondents had serious apprehensions and presented leading reservations as they had experienced such situations earlier.

***“Once a terrorism-related event occurs, the chance of secondary attack increases because of mass gathering of the crowd at TC. Besides that, the aggressive crowd (attendees) start agitation and clamoring. It is very difficult to concentrate proper management of the victims when you have the fear of another suicide attack on your mind with the presence of an angry and hostile crowd all around.” (Physician 13, Male)***

#### Subtheme 4(b): Lack of Disaster Management Content

Lack of materials related to disaster management in the curriculum of medical education as well as during the residency training period was also stated as a barrier. The respondents stated that they had no idea or adequate understandings of disaster management when they started their medical practice. Therefore, they had only one option to follow the established practices and to perform what is being practiced at their workplace.

***“The curriculum of medical education has nothing about disaster management. We are also unaware about disaster management during our residency training period. Disaster management is an important subject, and we must be informed and trained about it.” (Physician 15, Male)***

#### Subtheme 4(c): Lack of Drills and Hands-On Training

Our study respondents additionally mentioned that the lack of disaster-related training; seminars and workshops are potential barriers toward effective disaster management. Drills, mock exercises, and hands-on training sessions are essential to capacity building because as it increases the efficiency and effective management of emergencies and crisis situations.

***“The institute never has arranged any disaster management seminar or workshop, considering about disaster management training remains one step ahead. In such circumstances expecting disaster drills and mock exercises are beyond the imaginations.” (Physician 6, Female)***

### Theme 5: Suggestions and Recommendations

All physicians agreed and suggested that expansion of TC, enhancement of human resource, and increase logistics and supplies according to the requirements are needed. Furthermore, streamlining the line of instructions, provision of SOPs manual, plans, protocols, a checklist of TC, along with arranging training programs, exercises and systemically review and assessment of measures taken toward preparedness of the TC were key recommendations of the study respondents.

***“We need additional space for expansion, specialized healthcare professionals along with allied staff and adequate supplies. Moreover, the administration must focus on our skills development and capacity building through regular training and exercises.” (Physician 15, Female)***

## Discussion

Terrorism-related disasters not only results in to mass casualties but also produce massive economic loss (50). The terrorism-related attacks are aimed to instigate jeopardy, in turn, certain use of unlawful turmoil and rampage to produce apprehension, menacing, or intimidation (7). Terrorism-related attacks are not only aimed at targeting the victims but also sending a message of fear among communities (51). Hence, the population is confronted with developing a substantial number of long-term problems imposed by terrorism-related disasters (52). Data reveals that 18 - 50% of post-traumatic stress disorder (PTSD) were found prevalent among the victims of large-scale terrorism-related events (53). Besides this, terrorism is bilaterally associated with economic activity (54) and has the potential to adversely affect the economic growth (55), by marginalizing its modes of production and consumption globally (56). Additionally, the health care system is also under the siege of paramount multidimensional consequences of terrorism, even more susceptible to being a major stakeholder during its occurrence.

As discussed, the current study was aimed to assess the response and preparedness of physicians toward terrorism-related disaster management. We believe that the interviews extracted enough information that was able to answer the questions that were established earlier. The province of Balochistan has remained a leading victim of terrorism-related events for decades and the uncertainty turned the province into an undeclared warzone (57). Although significant reductions in terrorism-related deaths were reported across Pakistan in 2018, the number of terrorism-related deaths increased in Balochistan. Due to the strategic location of the province, the area specifically remained the worst victim of terrorism after 9/11, which fuelled the terrorism-related events (58). During this confrontation, the health care system of the province always remained at the front line in the provision of critical medical support and healthcare services to the victims of terrorism-related incidences. However, despite all the dedication, enthusiasm, and professional efforts of the healthcare system, the system of preparedness and response toward terrorism-related disasters was undefined ad that was to be explored in this current study.

Respondents of the current study highlighted that financial constraints, dearth of human resources, and poor healthcare system are the major barriers to ineffective management of terrorism-related disasters. We must remember that Pakistan's economy has paid both direct and indirect costs of approximately US $126.79 billion (7, 59) during the war against terror. Furthermore, terrorism negatively affected the flow of foreign investment, international trade, exports along with the local business in the country, which impacted lowered GDP (59). Pakistan is bearing a huge cost as government expenditures are diverted to counter-terrorism activities instead of valuable measures (54). Eventually, this diversion of expenditure overburdens the already deprived healthcare system as the policymakers are confronted with economic and financial constraints that impose restrictions on the provision of adequate healthcare services (60). However, even with all these limitations, the establishment of well-equipped TC, employment of proficient healthcare professionals, availability of logistic and medical support is evident of dedication while responding and preparing for a terrorism-related incidence.

Physicians are on the front lines while dealing with casualties or disasters (61). However, the foundation of medicine seems to be the opposite in the case of terrorism because where physicians blindly heal, terrorists blindly kill (62). Therefore terrorism-related events require additional and sophisticated medical assistance provided by healthcare professionals specifically the physicians (63). To effectively respond to mega disasters, physicians must be prepared to handle multi-casualty incidents within their own hospitals (64). However, while managing terrorism-related disasters, physician's training may be deficient; therefore, simulated training may be required in effective and adequate preparation and enhancement of confidence to respond to terrorism-related disasters (65). This requirement is also evident from our study where the respondents agreed about the lack of training and security measures which means that they are putting their own life and the safety of the patients at risk. Focusing the training and development while dealing with terrorism related disasters, physician education and experience is of considerable significance. Understanding disaster medicine and the healthcare system response in responding to terrorism-related events should be an indispensable part of the medical curriculum. Scott and colleagues reported that medical students highlighted that they have not received adequate training to respond to disasters (66) and the same is reflected in the notion of the current study respondents.

Management of disasters and other mass casualties such as suicide bombing, IEDs, road-side bombing, and mass shooting need concrete plans. The National Association of EMS Physicians (NAEMSP) recommends a substantial role of emergency medical services (EMS) in all phases of the disaster management cycle, including preparedness, response, and recovery (61). Additionally, American Medical Association (AMA) compiled an educational framework comprising 37 target capabilities after being reviewed by stakeholders and further reviewed by National Disaster Life Support Education Consortium (NDLSEC), with interest in disaster preparedness, professional education, and curriculum development (67). The World Association of Disaster Medicine has also emphasized the need for a more scientific approach to all aspects of disaster medicine for the effective preparedness and response to terrorism-related disasters (68). The dilemmas of the response activity and chances of deficiency in preparation cannot be overlooked (69). In line to these guidelines, physicians of the current study were willing to participate in practical training rather than classroom teaching which is in accordance with the proposed literature (70). Unfortunately, respondents of the current study were unaware of such guidelines and protocols. Physicians of our study revealed that they had been provided traditional education in the classrooms; they also elaborated that they are unaware of updated and standardized strategies. Moreover, performing routine medical practices requisite to the provision of treatment to the patients, realizing updated information is least focused. Referring to the discussion, a comprehensive reconsideration of the medical education curriculum is required for integrated standardization for future physicians, as well as condensed training and continuous medical education for practicing physicians is the only way to fill the gaps and improvement in disaster management competencies. If patients are the first victims of medical error, then physicians are termed as the “second victims” as they often experience feelings of distress, guilt, shame, and depression in response (71). The internationally accepted competency-oriented framework of physicians' education provides a thorough understanding of emergency preparedness and response, which it appeared to be missing in the current healthcare system of Pakistan.

However, our study respondents agreed that for effective management of unexpected catastrophic situations, disaster preparedness is worth of paramount significance; they also reported that, unfortunately, there is a dearth of training in the implementation of proficient disaster management. Our findings are supported by Barbosa through a survey from 224 hospital emergency departments in four North-western states, and the author identified deficiencies in preparedness involving physician training while managing terrorism-related disasters (69). It is a matter of prime concern that physicians are aware of adequate and efficient triaging, emergency management, mitigation, and rehabilitation of the victims back to their normal life. Additionally, physicians should also be trained in the provision of care, psychosocial support, and most importantly, carrying out limb, organ, and life-saving treatment to the victims. In consideration of its importance, the Office of the Assistant Secretary for Preparedness and Response (ASPR) and the Department of Defence (DoD) recommended that working together with partners to prioritize and develop examples of competency-based knowledge and skills must be included in job descriptions for public health, healthcare, behavioral health, and other national health security personnel and inter-professional health and supporting teams (72). But unfortunately, physicians, the respondents of our current study, were unaware of basic elements of disaster management such as triage principles, inter-coordination, reintegration, recuperation that are evidently ratiocinated the immediate requirement of training and development of physicians, particularly in the predicament of terrorism-related disaster management.

Physicians as the main pillar and leading arm of the health care system are required to acquire more formal education and training in both preparedness and response to be credentialed to assist (63). Revealed research recommended that disaster medicine be augmented either in the curriculum of undergraduate medical schools or in postgraduate university-based programs additionally in the continuing medical educations programs (73). This enables the physicians to overcome the anarchic circumstances. Our qualitative analysis reflected the need for the incorporation of emergency/disaster medicine in the regular curriculum of medical schools in Pakistan, along with extensive disaster management training supported by the World Health Organization.

## Conclusion

Physicians are the leading arm of the healthcare system and, as team player, has a supervisory role in managing terrorism-related disasters. The competencies, skills-set, and expertise required to deal with a terrorism-related disaster are well recognized but are deficient due to several considerations. Interpreting the physicians' perspectives through the extracted themes revealed reconsideration and integration of standardized curriculum of medical education particularly relevant to terrorism-related disaster management. Additionally, regular training and table-top exercises for practicing physicians were also emphasized that will encourage effective and confident responses in disaster management. Finally, conferences, seminars CME programs are required to share the current experiences and to meet harmonization in the latest practices adopted globally to counter terrorism-related disasters.

## Limitations

The limited information from the study settings made it hard to cross compare the data extracted from this study. Furthermore, being qualitative in nature the generalizability is always an issue. We recommend conducting a quantitative study based on the identified theme with a larger group of physicians to get a better insight of preparedness and response toward terrorism-related disaster events.

## Data Availability Statement

The original contributions presented in the study are included in the article/Supplementary Material, further inquiries can be directed to the corresponding author/s.

## Ethics Statement

The studies involving human participants were reviewed and approved by Faculty of Pharmacy and Health Sciences, University of Balochistan, Quetta. The patients/participants provided their written informed consent to participate in this study.

## Author Contributions

FK, Z-e-H, NB, and MS conducted the literature review and developed the interview protocol and the guide. FK conducted the interviews while ZI and AR monitored the process as observers. NS, SA, and SH analyzed and drafted the manuscript, which was subject to critical revision by QI, TH, AA, and RI. FS supervised the study. AA contributed in analysis, theme generation, and write up of the initial draft. All authors read and approved the final manuscript and contributed equally.

## Conflict of Interest

The authors declare that the research was conducted in the absence of any commercial or financial relationships that could be construed as a potential conflict of interest.

## Publisher's Note

All claims expressed in this article are solely those of the authors and do not necessarily represent those of their affiliated organizations, or those of the publisher, the editors and the reviewers. Any product that may be evaluated in this article, or claim that may be made by its manufacturer, is not guaranteed or endorsed by the publisher.

## References

[1] Shafiq F Ahsan K. Knowledge management for disaster scenario: an exploratory study. Res J Recent Sci. (2013) 2:61–6. 26750812

[2] Al-Dahash H Kulatunga U Al-Dehesh A. Disaster response management stemming from war operation and terrorism in Iraq. In: 12th International Post-Graduate Research Conference, 10-12 June. MediaCity (2015).

[3] Putra A Petpichetchian W. Public health nurses' roles and competencies in disaster management. Nurse Media J Nurs. (2011) 1:1–14. 10.14710/nmjn.v1i1.742

[4] Nofal A Alfayyad I Khan A Al Aseri Z Abu-Shaheen A. Knowledge, attitudes, and practices of emergency department staff towards disaster and emergency preparedness at tertiary health care hospital in central Saudi Arabia. Saudi Med J. (2018) 39:1123–9. 10.15537/smj.2018.11.23026 30397712 PMC6274652

[5] Ahmed Z. Disaster risks and disaster management policies and practices in Pakistan: a critical analysis of Disaster Management Act 2010 of Pakistan. Int J Disaster Risk Reduct. (2013) 4:15–20. 10.1016/j.ijdrr.2013.03.003

[6] Tolan GM Soliman OS. An experimental study of classification algorithms for terrorism prediction. Int J Knowl Eng. (2015) 1:107–12. 10.7763/IJKE.2015.V1.18

[7] Zakaria M Jun W Ahmed H. Effect of terrorism on economic growth in Pakistan: an empirical analysis. Econ Res. (2019) 32:1794–812. 10.1080/1331677X.2019.1638290

[8] Ding F Ge Q Jiang D Fu J Hao M. Understanding the dynamics of terrorism events with multiple-discipline datasets and machine learning approach. PLoS ONE. (2017) 12:e0179057. 10.1371/journal.pone.0179057 28591138 PMC5462416

[9] Magnus D Khan MA Proud WG. Epidemiology of civilian blast injuries inflicted by terrorist bombings from 1970-2016. Defence Technol. (2018) 14:469–76. 10.1016/j.dt.2018.07.014

[10] Santos C El Zahran T Weiland J Anwar M Schier J. Characterizing chemical terrorism incidents collected by the global terrorism database, 1970-2015. Prehosp Disaster Med. (2019) 34:385–92. 10.1017/S1049023X19004539 31280729 PMC6800028

[11] Czinkota MR Knight G Liesch PW Steen J. Terrorism and international business: a research agenda. J Int Bus Stud. (2010) 41:826–43. 10.1057/jibs.2010.1214960137

[12] Edwards DS Mcmenemy L Stapley SA Patel HDL Clasper JC. 40 years of terrorist bombings–a meta-analysis of the casualty and injury profile. Injury. (2016) 47:646–52. 10.1016/j.injury.2015.12.021 26830126

[13] Akhmat G Zaman K Shukui T Sajjad F. Exploring the root causes of terrorism in South Asia: everybody should be concerned. Qual Quant. (2014) 48:3065–79. 10.1007/s11135-013-9941-2

[14] Azhar A Malik MN Muzaffar A. Social network analysis of Army Public School Shootings: need for a unified man-made disaster management in Pakistan. Int J Disaster Risk Reduct. (2019) 34:255–64. 10.1016/j.ijdrr.2018.11.024

[15] Shahzad U Sarwar S Farooq MU Qin F. USAID official development assistance and counter terrorism efforts: pre and post 9/11 analysis for South Asia. Socioecon Plann Sci. (2020) 69:100716. 10.1016/j.seps.2019.06.001

[16] Zafar H Jawad A Shamim MS Memon AA Hameed A Effendi MS. Terrorist bombings: medical response in a developing country. J Pak Med Assoc. (2011) 61:561–6. 22204211

[17] Rosenfeld JV Mitra B Smit DV Fitzgerald MC Butson B Stephenson M. Preparedness for treating victims of terrorist attacks in Australia: learning from recent military experience. Emerg Med Australasia. (2018) 30:722–4. 10.1111/1742-6723.13091 29740959

[18] Shahbaz M Shabbir MS Malik MN Wolters ME. An analysis of a causal relationship between economic growth and terrorism in Pakistan. Econ Model. (2013) 35:21–9. 10.1016/j.econmod.2013.06.031

[19] Najaf K Najaf R. Impact of cost of war against terrorism on the different determents of FDI of Pakistan. Int J Res Granthaalayah. (2016) 4:156–68. 10.29121/granthaalayah.v4.i5.2016.2691

[20] Rauf S Mehmood R Rauf A Mehmood S. Integrated model to measure the impact of terrorism and political stability on FDI inflows: empirical study of Pakistan. Int J Econ Finance. (2016) 8:1–7. 10.5539/ijef.v8n4p1

[21] Shahzad SJH Zakaria M Rehman MU Ahmed T Fida BA. Relationship between FDI, terrorism and economic growth in Pakistan: pre and post 9/11 analysis. Soc Indic Res. (2016) 127:179–94. 10.1007/s11205-015-0950-5

[22] Edwards B Issa F Voskanyan A Ciottone G. Counter-terrorism medicine: creating a medical initiative mandated by escalating asymmetric attacks. Prehosp Disaster Med. (2020) 35:595–8. 10.1017/S1049023X2000103X 32792026

[23] Zaheer H. Blood management in disaster situations in Pakistan. Sci Ser. (2012) 7:1–5. 10.1111/j.1751-2824.2012.01550.x

[24] Grossman D Khalil U Ray A. Terrorism and early childhood health outcomes: evidence from Pakistan. Soc Sci Med. (2019) 237:112453. 10.1016/j.socscimed.2019.112453 31442823

[25] Rafiq L Blaschke T. Disaster risk and vulnerability in Pakistan at a district level. Geomat Nat Hazards Risk. (2012) 3:324–41. 10.1080/19475705.2011.626083

[26] Maqbool A Afzal F Razia A. Disaster mitigation in Urban Pakistan using agent based modeling with GIS. Int J Geo Inform. (2020) 9:203. 10.3390/ijgi9040203

[27] Umer M Sepah YJ Shahpurwala MM Zafar H. Suicide bombings: process of care of mass casualties in the developing world. Disasters. (2009) 33:809–21. 10.1111/j.1467-7717.2009.01110.x 19624704

[28] Kitzinger J. Qualitative research: introducing focus groups. Br Med J. (1995) 311:299–302. 10.1136/bmj.311.7000.299 7633241 PMC2550365

[29] Berg BL Lune H Lune H. Qualitative Research Methods for the Social Sciences. Vol 5. Boston, MA: Pearson (2004).

[30] Krueger RA. Focus Groups: A Practical Guide for Applied Research. California, CA: Sage Inc ISBN: 1412969476 (2009).

[31] Stewart DW Shamdasani PN. Focus Groups: Theory and Practice. Vol. 20. California, CA: Sage Publications (2014).

[32] Mullen PD Reynolds R. The potential of grounded theory for health education research: linking theory and practice. Health Educ Behav. (1978) 6:280–94. 10.1177/109019817800600302 752665

[33] Entwistle VA Renfrew MJ Yearley S Forrester J Lamont T. Lay perspectives: advantages for health research. Br Med J. (1998) 316:463–6. 10.1136/bmj.316.7129.463 9492683 PMC2665609

[34] Shahzad F Saleem F Iqbal Q Haque N Haider S Salman M. Cross-sectional assessment of health literacy among hypertensive community of Quetta City, Pakistan. Biomed J Sci Tech Res. (2018) 4:1–9. 10.26717/BJSTR.2018.11.002141

[35] Brace-Govan J. Issues in snowball sampling: the lawyer, the model and ethics. Qual Res J. (2004) 4:52.

[36] Labrague L Hammad K Gloe D McEnroe-Petitte D Fronda D Obeidat A. Disaster preparedness among nurses: a systematic review of literature. Int Nurs Rev. (2018) 65:41–53. 10.1111/inr.12369 28295314

[37] Wetta-Hall R Fredrickson DD Ablah E Cook DJ Molgaard CA. Knowing who your partners are: terrorism-preparedness training for nurses. J Continuing Educ Nurs. (2006) 37:106–12. 10.3928/00220124-20060301-03 18814391

[38] Rose MA Larrimore KL. Knowledge and awareness concerning chemical and biological terrorism: continuing education implications. J Continuing Educ Nurs. (2002) 33:253–8. 10.3928/0022-0124-20021101-05 12442873

[39] Veenema TG. Disaster Nursing and Emergency Preparedness. New York, NY: Springer Publishing Company (2018).

[40] Cox CW. Manmade disasters: a historical review of terrorism and implications for the future. Online J Issues Nurs. (2008) 13. 10.3912/OJIN.Vol13No01PPT04

[41] Kallio H Pietilä AM Johnson M Kangasniemi M. Systematic methodological review: developing a framework for a qualitative semi-structured interview guide. J Adv Nurs. (2016) 72:2954–65. 10.1111/jan.13031 27221824

[42] Morris A. A Practical Introduction to In-Depth Interviewing. London: Sage (2015).

[43] Voutsina C. A practical introduction to in-depth interviewing. Int J Res Method Educ. (2018) 41:123–4. 10.1080/1743727X.2017.1419693

[44] Saunders B Sim J Kingstone T Baker S Waterfield J Bartlam B. Saturation in qualitative research: exploring its conceptualization and operationalization. Qual Quant. (2018) 52:1893–907. 10.1007/s11135-017-0574-8 29937585 PMC5993836

[45] Nelson J. Using conceptual depth criteria: addressing the challenge of reaching saturation in qualitative research. Qual Res. (2017) 17:554–70. 10.1177/1468794116679873

[46] Guest G MacQueen KM Namey EE. Introduction to applied thematic analysis. Appl Them Anal. (2012) 3:20. 10.4135/9781483384436.n1

[47] Anderson R. Thematic Content Analysis (TCA). Descriptive Presentation of Qualitative Data. (2007). Available online at: http://rosemarieanderson.com/wp-content/uploads/2014/08/ThematicContentAnalysis.pdf (accessed June 15, 2021).

[48] Vaismoradi M Turunen H Bondas T. Content analysis and thematic analysis: implications for conducting a qualitative descriptive study. Nurs Health Sci. (2013) 15:398–405. 10.1111/nhs.12048 23480423

[49] Edhlund B McDougall A. Nvivo 12 Essentials. Stallarholmen: FORM & KUNSKAP AB. ISBN: 1387749498 (2019).

[50] Ismail A Amjad S. Determinants of terrorism in Pakistan: an empirical investigation. Econ Model. (2014) 37:320–31. 10.1016/j.econmod.2013.11.012

[51] Möller-Leimkühler AM. Why is terrorism a man's business? CNS Spectr. (2018) 23:119–28. 10.1017/S1092852917000438 28766471

[52] Rauf A Siddique HMA Saleem Q Sidra S. Terrorism and international tourism nexus: evidence from Pakistan. Int J Econ Financial Issues. (2020) 10:387. 10.32479/ijefi.10674

[53] North CS Pfefferbaum B Kawasaki A Lee S Spitznagel EL. Psychosocial adjustment of directly exposed survivors 7 years after the Oklahoma City bombing. Compr Psychiatry. (2011) 52:1–8. 10.1016/j.comppsych.2010.04.003 21220059 PMC3039884

[54] Saleem Q Sidra S Rauf A Siddique HMA. Impact of terrorism on economic growth in South Asian country. Int J Econ Financial Issues. (2020) 10:185. 10.32479/ijefi.9699

[55] Bayar Y Gavriletea MD. Peace, terrorism and economic growth in Middle East and North African countries. Qual Quant. (2018) 52:2373–92. 10.1007/s11135-017-0671-8

[56] Mohamed H Jebli MB Youssef SB. Renewable and fossil energy, terrorism, economic growth, and trade: evidence from France. Renew Energy. (2019) 139:459–67. 10.1016/j.renene.2019.02.096

[57] Niaz B Hassan A Irtaza S. Role of media in minimizing religious extremism and ethnic instability in Balochistan, Pakistan. Glob Reg Rev. (2020) 4:12–20. 10.31703/grr.2020(V-IV).02

[58] Syed SH Saeed L Martin RP. Causes and incentives for terrorism in Pakistan. J Appl Security Res. (2015) 10:181–206. 10.1080/19361610.2015.1004606

[59] Khan NH Ju Y Hassan ST. Modeling the impact of economic growth and terrorism on the human development index: collecting evidence from Pakistan. Environ Sci Pollut Res. (2018) 25:34661–73. 10.1007/s11356-018-3275-5 30324364

[60] Zaidi SA Bigdeli M Langlois EV Riaz A Orr DW Idrees N. Health systems changes after decentralisation: progress, challenges and dynamics in Pakistan. BMJ Global Health. (2019) 4:e001013. 10.1136/bmjgh-2018-001013 30805206 PMC6357909

[61] Catlett CL Jenkins JL Millin MG. Role of emergency medical services in disaster response: resource document for the National Association of EMS Physicians position statement. Prehosp Emerg Care. (2011) 15:420–5. 10.3109/10903127.2011.561401 21480774

[62] Lederman Z Voo T. Should we prioritise victims over terrorists in medical triage? BMJ Military Health. (2019) 165:266–9. 10.1136/jramc-2018-001009 30127066

[63] Kumar A Weibley E. Disaster management and physician preparedness. South Med J. (2013) 106:17–20. 10.1097/SMJ.0b013e3827c5c5b 23263308

[64] Russo RM Galante JM Jacoby RC Shatz DV. Mass casualty disasters: who should run the show? J Emerg Med. (2015) 48:685–92. 10.1016/j.jemermed.2014.12.069 25837230

[65] Pintea M Dahl Grove D. Primary care physicians: an untapped resource for disaster response. Curr Treat Opt Pediatr. (2019) 5:276–83. 10.1007/s40746-019-00164-5

[66] Scott LA Carson DS Greenwell IB. Disaster 101: a novel approach to disaster medicine training for health professionals. J Emerg Med. (2010) 39:220–6. 10.1016/j.jemermed.2009.08.064 20079997

[67] Subbarao I Lyznicki JM Hsu EB Gebbie KM Markenson D Barzansky B. Consensus-based educational framework and competency set for the discipline of disaster medicine and public health preparedness. Disaster Med Public Health Prep. (2008) 2:57–68. 10.1097/DMP.0b013e31816564af 18388659

[68] Ingrassia PL Prato F Geddo A Colombo D Tengattini M Calligaro S. Evaluation of medical management during a mass casualty incident exercise: an objective assessment tool to enhance direct observation. J Emerg Med. (2010) 39:629–36. 10.1016/j.jemermed.2009.03.029 19570646

[69] Barbosa F. Emergency health care system and its role in national disasters. J Human Insights. (2018) 2:14–20. 10.22034/jhi.2018.61425

[70] Zhiheng Z Caixia W Jiaji W Huajie Y Chao W Wannian L. The knowledge, attitude and behavior about public health emergencies and the response capacity of primary care medical staffs of Guangdong Province, China. BMC Health Serv Res. (2012) 12:1–9. 10.1186/1472-6963-12-338 23009075 PMC3489590

[71] Gregory J de Lepinau J de Buyer A Delanoy N Mir O Gaillard R. The impact of the Paris terrorist attacks on the mental health of resident physicians. BMC Psychiatry. (2019) 19:1–8. 10.1186/s12888-019-2058-y 30791878 PMC6385411

[72] Burkle FM. The development of multidisciplinary core competencies: the first step in the professionalization of disaster medicine and public health preparedness on a global scale. Disaster Med Public Health Prep. (2012) 6:10–2. 10.1001/dmp.2012.3 22490931

[73] Naser WN Saleem HB. Emergency and disaster management training; knowledge and attitude of Yemeni health professionals-a cross-sectional study. BMC Emerg Med. (2018) 18:1–12. 10.1186/s12873-018-0174-5 30081832 PMC6091203

