# Supplementary material for: Physicians' Response and Preparedness of Terrorism-Related Disaster Events in Quetta City, Pakistan: A Qualitative Inquiry
Source: Front Public Health. 2022 Jun 27;10:912762. doi: 10.3389/fpubh.2022.912762 (PMC9271701; doi:10.3389/fpubh.2022.912762)
Supplement: Supplementary file 1 [file Table_1.DOCX]

**QUALITATIVE GUIDE**

Age: ___________ Gender: ____________ Education: ______________________________

Overall Experience: __________________ Experience at Trauma Centre: _______________

Current Position: ____________________ Special certification of D.M__________________

**Response Phase**

What do you think about your knowledge of terrorism related disaster management?

Are you aware about the hospital plans, protocols and laws that address to terrorism related disaster response and preparedness?

Plans:

Protocols:

Laws:

Q-1: Have you ever experienced to deal terrorism related disaster? Yes________ No_______ (If yes)

1. Nature and time period of the disaster event: ____________________________________

Bomb Blast________ Suicidal Attack__________ Assassination_______ Other________

1. What was your initial response?

Physical ________________________________________________________________

Emotional ______________________________________________________________

1. How did you come to know about the disaster event (source of information)
2. What is the information system to call in case of terrorism related disaster event?

1. After How much time you reached at your work place?
2. Usually how much time takes place to complete the team of professionals?
3. Are you aware about the line of instruction during the course of terrorism related disaster event?
4. What do you think about the flow of communication and flow of instruction during the response of terrorism related event at your work place?
5. After How much time you received the victims of terrorist event occurred?
6. What was environment during terrorism related disaster event?
7. What was your plan to triage the victims of terrorist event?

How many patients you can handle at a time

1. How do you compare your response to nature of disaster
2. How did u find the teamwork
3. How well you responded in terms of healthcare professional as a team

Surgeons

Nurses

Pharmacists

Other supportive staff

1. Do you think the staffs were sufficient to deal the event or to be needed more staff to overcome properly?
2. In your view is your response was professionally sufficient to overcome the disaster event?

Yes ____________ If Yes No _______________ If No

What do think about your performance in terms of? Reasons:

Staff ______________________________________ 1: _____________________

Space _____________________________________ 2: _____________________

Medicine ___________________________________ 3: _____________________

Surgical and disposable items ______________________ 4: _____________________

Surgical equipments ______________________________ 5: _____________________

Laboratory investigations __________________________ 6: _____________________

1. Did you felt to shift or refer the victims to somewhere else due to over burden of patients or due to unavailability of beds and other facilities?
2. What was your overall response to disaster

Professional ________________________ unprofessional _________________________

1. According to your view what are the limitations in response to terrorism related disaster
2. What are your suggestions in improving response to terrorism related disaster?

**PREPARDNESS**

1: Do you have any specific qualification related to disaster management?

2: Have you attended any training regarding disaster management?

3: Have you attended any seminar regarding disaster management?

4: Have you attended any workshop regarding disaster management?

5: Have you attended any exercise regarding disaster management?

6: Have you attended any disaster drill regarding disaster management?

7: Have you provided a disaster plan from your administration?

8: Have you provided any manual or protocol book from your administration?

9: Have your provided standard operating procedures regarding disaster management by your administration?

10: Have you provided communication flow chart by your administration?

11: Have you aware about inter-organizational coordination?

12: Do you think you are prepared to terrorism related disaster?

13: According to your view how well you are prepared to terrorism related disaster?

If good how _________________________________________________________________

If not good how ______________________________________________________________

14: In normal days what are your activities regarding preparedness to terrorism related disaster?

Handling it: _________________________________________________________________

Pre-disaster drills: ____________________________________________________________

Disaster management protocols: _________________________________________________

15: Does the hospital have plan for disaster drills?

Seminars / workshops

16: In your view is the support from hospital administration sufficient or not?

17: Do you think your current level of preparedness is sufficient to address to terrorism related disaster?

Yes_______________________________ No ________________________________

__________________________________ ____________________________________

__________________________________ ____________________________________

__________________________________ ____________________________________

18: According to your view what are the limitations in preparedness to terrorism related disaster at your work place?

19: What do suggest regarding improvement in the preparedness to terrorism related disaster?
